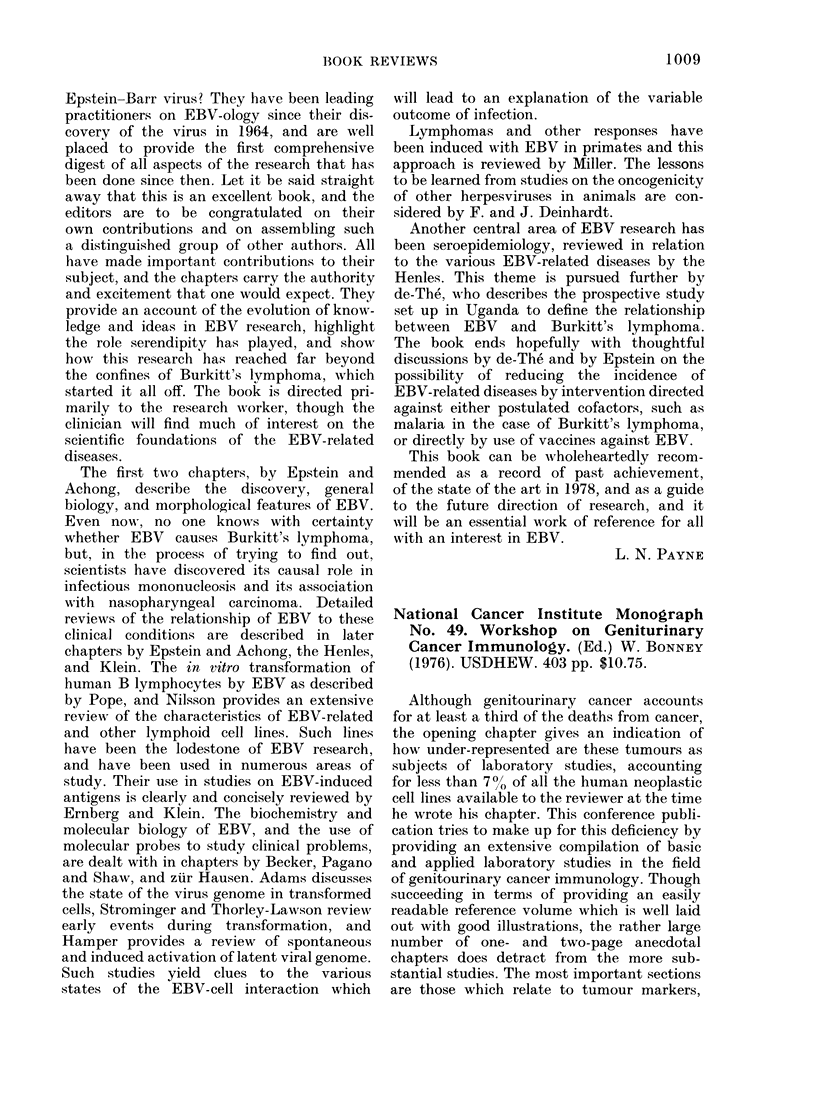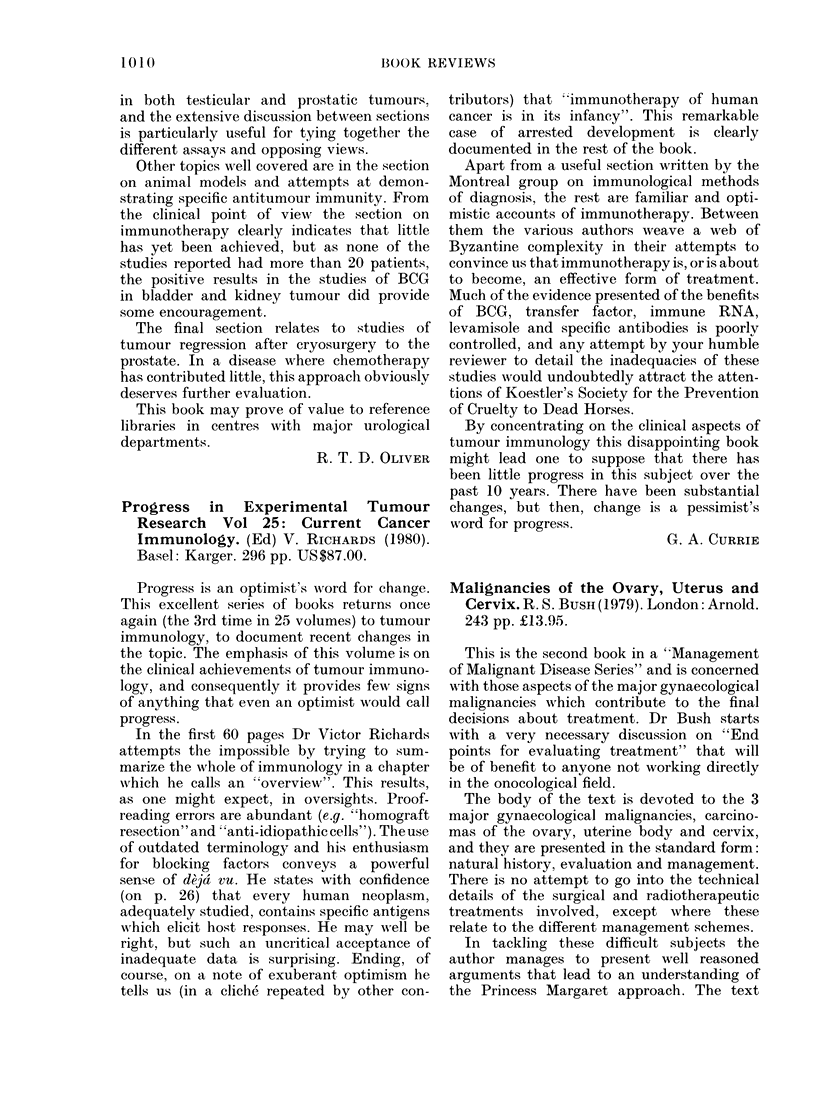# National Cancer Institute Monograph No. 49. Workshop on Geniturinary Cancer Immunology

**Published:** 1980-06

**Authors:** R. T. D. Oliver


**National Cancer Institute Monograph No. 49. Workshop on Geniturinary Cancer Immunology.** (Ed.) W. BONNEY (1976). USDHEW. 403 pp. $10.75.

Although genitourinary cancer accounts for at least a third of the deaths from cancer, the opening chapter gives an indication of how under-represented are these tumours as subjects of laboratory studies, accounting for less than 7% of all the human neoplastic cell lines available to the reviewer at the time he wrote his chapter. This conference publication tries to make up for this deficiency by providing an extensive compilation of basic and applied laboratory studies in the field of genitourinary cancer immunology. Though succeeding in terms of providing an easily readable reference volume which is well laid out with good illustrations, the rather large number of one- and two-page anecdotal chapters does detract from the more substantial studies. The most important sections are those which relate to tumour markers, in both testicular and prostatic tumours, and the extensive discussion between sections is particularly useful for tying together the different assays and opposing views.

Other topics well covered are in the section on animal models and attempts at demonstrating specific antitumour immunity. From the clinical point of view the section on immunotherapy clearly indicates that little has yet been achieved, but as none of the studies reported had more than 20 patients, the positive results in the studies of BCG in bladder and kidney tumour did provide some encouragement.

The final section relates to studies of tumour regression after cryosurgery to the prostate. In a disease where chemotherapy has contributed little, this approach obviously deserves further evaluation.

This book may prove of value to reference libraries in centres with major urological departments.

R. T. D. Oliver